# The roles of acquired and innate immunity in human T-cell leukemia virus type 1-mediated diseases

**DOI:** 10.3389/fmicb.2012.00323

**Published:** 2012-09-03

**Authors:** Mari Kannagi, Atsuhiko Hasegawa, Ayako Takamori, Shuichi Kinpara, Atae Utsunomiya

**Affiliations:** ^1^Department of Immunotherapeutics, Graduate School, Tokyo Medical and Dental UniversityTokyo, Japan; ^2^Department of Hematology, Imamura Bun-in HospitalKagoshima, Japan

**Keywords:** adult T-cell leukemia, cytotoxic T lymphocytes, human T-cell leukemia virus type 1, HTLV-1-associated myelopathy/tropical spastic paraparesis, type-I interferon

## Abstract

Human T-cell leukemia virus type 1 (HTLV-1) causes adult T-cell leukemia (ATL) and HTLV-1-associated myelopathy/tropical spastic paraparesis in small subsets of HTLV-1 carriers. HTLV-1-specific T-cell responses play critical roles in anti-viral and anti-tumor host defense during HTLV-1 infections. Some HTLV-1 carriers exhibit selective loss or anergy of HTLV-1-specific T-cells at an asymptomatic stage. This is also observed in ATL patients and may therefore be an underlying risk factor of ATL in combination with elevated proviral loads. HTLV-1-specific T-cells often recognize the viral oncoprotein Tax, indicating expression of Tax protein *in vivo*, although levels of HTLV-1 gene expression are known to be very low. A type-I interferon (IFN) response can be induced by HTLV-1-infected cells and suppresses HTLV-1 expression *in vitro*, suggesting a role of type-I IFN response in viral suppression and pathogenesis *in vivo*. Both acquired and innate immune responses control the status of HTLV-1-infected cells and could be the important determinants in the development of HTLV-1-mediated malignant and inflammatory diseases.

## INTRODUCTION

Human T-lymphotropic virus type I (HTLV-1) causes adult T-cell leukemia (ATL) (Hinuma et al., 1981; Uchiyama, 1997) and HTLV-1-associated myelopathy/tropical spastic paraparesis (HAM/TSP; Gessain et al., 1985; Osame et al., 1986) in a subset of infected individuals. While ATL is a malignant lymphoproliferative disease, HAM/TSP presents as a chronic inflammatory neurodegenerative disease. It is not known how one virus can cause two vastly different diseases. Since no disease-specific difference among HTLV-1 strains have been identified (Daenke et al., 1990; Kinoshita et al., 1991), the different pathogenic consequences must be attributed to host factors. Indeed, the two diseases usually occur in different populations of HTLV-1 carriers. Identification of the determinant factors may allow the prediction of disease risks and also the development of prophylactic and therapeutic strategies.

One of the factors that are known to differ between ATL and HAM/TSP patients is the strength of HTLV-1-specific T-cell responses. T-cell responses are activated in HAM/TSP patients, but are weak in ATL patients, and are thus considered to be one of the most important determinants of the disease manifestation. Many investigators, including us, have been investigating HTLV-1-specific cytotoxic T lymphocyte (CTL) responses, and demonstrated the importance of these CTLs on anti-viral and anti-tumor surveillance in HTLV-1-infected hosts (Jacobson et al., 1990; Bangham and Osame, 2005; Kannagi et al., 2005). Based on these studies we concluded that a reduced HTLV-1-specific T-cell response can be an underlying risk factor for the development of ATL.

Another difference between HAM/TSP and ATL patients is the level of HTLV-1 gene expression. Although HTLV-1 mRNA levels are generally low *in vivo*, they are slightly higher in HAM/TSP patients compared with asymptomatic HTLV-1 carriers (ACs; Yamano et al., 2002). The mechanism causing low levels of HTLV-1 gene expression *in vivo* remains unknown. However, we recently demonstrated that HTLV-1 expression is suppressed by non-lymphoid stromal cells through type-I interferon (IFN), indicating that innate immune responses can be another host determinant for HTLV-1-induced diseases (Kinpara et al., 2009). So far there have only been a limited number of studies reporting a type-I IFN response during HTLV-1 infection.

The status of HTLV-1 expression is critical for host immune responses and viral pathogenesis. In particular, HTLV-1 Tax is a multipotent protein that is a main target of T-cell immunity (Jacobson et al., 1990; Kannagi et al., 1991) and can activate NF-κB, a characteristic transcriptional factor in ATL cells and a strong inducer of inflammatory cytokines (Yoshida, 2001; Jeang et al., 2004; Grassmann et al., 2005). The status of Tax expression *in vivo* has been controversial but would be an extremely important factor to measure in order to understand the mechanism of disease development in HTLV-1 infection. Expression of HTLV-1 basic leucine zipper factor (HBZ) encoded by the minus-strand HTLV-1 genome is also an important factor for viral pathogenesis as HBZ elicits indirect effects on tumor development and inflammation (Satou et al., 2011).

In this review, we aim to understand the conflicting findings that have been reported in regard to HTLV-1 expression *in vivo*. We then describe recent findings that add to the knowledge about well-characterized host T-cell responses, followed by a description of the mechanisms that control viral expression. We finally discuss the relationship between HTLV-1 expression, host immune response, and HTLV-1-mediated malignant and inflammatory diseases.

## STATUS OF HTLV-1 EXPRESSION *IN VIVO*

**FIGURE 1:**
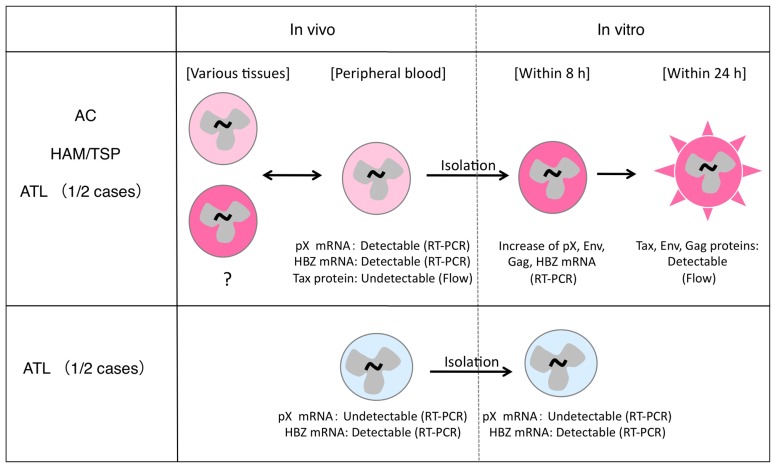
**Different status of HTLV-1 expression in infected cells *in vivo* and *in vitro*.** HTLV-1-infected cells express viral mRNA in the peripheral blood and can express viral proteins in a short-term culture *in vitro* (top). This phenomenon is observed in most HTLV-1-infected individuals (top), but not in 1/2 cases of ATL (bottom).

The understanding of HTLV-1 expression *in vivo* has caused much confusion, largely owing to two reasons: (a) HTLV-1 proteins are not detectable in infected cells in the peripheral blood of HTLV-1 carriers; (b) two types of ATL cases exist, and while HTLV-1 expression in ATL cells is conserved in some cases, this expression is lost in other cases (**Figure 1**).

### EXPRESSION OF HTLV-1 PROTEINS *IN VIVO*

HTLV-1 mRNA but not proteins are detectable in the peripheral blood mononuclear cells (PBMCs) of HTLV-1-infected individuals including ATL patients (Kinoshita et al., 1989). The presence of HTLV-1 mRNA was also reported in other tissues such as muscle in myositis patients (Tangy et al., 1995; Ozden et al., 2004) or the spinal cord in HAM/TSP patients (Lehky et al., 1995). Therefore, HTLV-1 gene expression does occur *in vivo* at least at a transcriptional level. Furthermore, based on the findings that HTLV-1-infected individuals maintain serum antibodies directed to the HTLV-1 structural Env and Core proteins as well as Tax-specific T-cells, HTLV-1 expression must be occurring also at a protein level *in vivo*. This notion is further supported by the emergence of Tax-specific CTL responses in ATL patients who received hematopoietic stem cells from HTLV-1-negative donors (Harashima et al., 2004, 2005). In these cases, the CTL responses are presumably triggered by the *de novo* exposure of donor-derived T-cells to Tax antigen in the recipients, and resemble an acute infection. These findings suggest that the sensitivity of T-cells recognizing HTLV-1 antigen may be much higher than the detection by serological means such as flow cytometry or immunoblotting, which are dependent on antibody binding. The conflicting arguments concerning HTLV-1 expression might thus continue until more sensitive protein detection methods are developed.

### TWO TYPES OF ATL WITH OR WITHOUT HTLV-1 EXPRESSION

HTLV-1 expression in ATL cells immediately after isolation from peripheral blood is very low, and becomes significantly induced only after *in vitro* cultivation (Hinuma et al., 1982). This phenomenon is observed in about half of the ATL cases, regardless of the severity of the disease (Kurihara et al., 2005). A similar induction of viral expression after *in vitro* culture has also been observed in PBMCs from HAM/TSP patients and ACs (Hanon et al., 2000). Recent analysis using quantitative RT-PCR methods confirmed this phenomenon in PBMCs from both ATL and HAM/TSP patients. The data further showed that levels of Tax/Rex mRNA were increased as early as 4 h after initiation of culture, and peaked at 8 h, followed by an increase in Env, Gag/Pol, and other mRNAs (Rende et al., 2011). This finding is consistent with the critical roles of Tax and Rex proteins for viral expression through transcriptional transactivation, regulation of RNA splicing, and nuclear export of the mRNAs, which were described in previous studies (Yoshida, 2001; Younis and Green, 2005). The rapid induction of viral expression in culture further suggests the presence of a common mechanism transiently suppressing HTLV-1 expression *in vivo*, irrespective of the disease.

In the remaining half of the ATL cases, however, such viral induction does not occur, even after *in vitro* culture. This may be due to genetic and epigenetic changes in the viral genome (Tamiya et al., 1996; Takeda et al., 2004). The malignant phenotype of ATL cells in these cases is presumably attributed to other mechanisms acquired at additional steps of leukemogenesis, independently of HTLV-1 expression.

### EXPRESSION OF HBZ IN INFECTED CELLS

In uncultured PBMCs from HTLV-1-infected individuals, expression of the HTLV-1 genome is suppressed as noted above, whereas mRNA of HBZ, the minus-strand HTLV-1-encoded gene, is continuously expressed, irrespective of the disease (Satou et al., 2006). Transcription of HBZ in the absence of Tax implies an indispensable role of HBZ in HTLV-1-infected cells. Interestingly, mice carrying an HBZ transgene under the control of the CD4 promoter often develop lymphoproliferative disease with frequent Foxp3 expression and inflammatory skin lesions (Satou et al., 2011). These features partly resemble the characteristics of ATL.

However, expression of HBZ at a protein level is still controversial. In a study analyzing mRNA kinetics during the initial culture of PBMC from infected individuals, Tax/Rex and other positive-strand transcripts were promptly exported to the cytoplasm after transcriptional induction, while HBZ mRNA was mostly retained in the nucleus (Rende et al., 2011). In addition, HBZ-specific CTLs induced in human leukocyte antigen (HLA)-A2-transgenic mice lysed HBZ peptide-pulsed HLA-A2-positive target cells but not HTLV-1-infected HLA-A2-positive cells (Suemori et al., 2009). These observations suggest that expression of HBZ at a protein level in HTLV-1-infected cells might be limited, even though substantial amounts of HBZ mRNA are expressed. Nevertheless, the presence of T-cells responding to HBZ peptides have been reported in HAM/TSP patients at a low frequency, implying a small amount of HBZ protein synthesis *in vivo* (Hilburn et al., 2011).

### HTLV-1 EXPRESSION IN HAM/TSP PATIENTS

HTLV-1 expression is detectable at the transcriptional, but not the protein level in uncultured PBMCs, and such basal levels of mRNA differ among diseases. An early study showed that the pX mRNA/DNA ratio was lower in ATL patients compared with ACs or HAM/TSP patients (Furukawa et al., 1995). This might partly reflect that in 50% of ATL cases the cells lost viral gene expression, as mentioned above. A recent study using a real-time quantitative PCR analysis indicated that HTLV-1 mRNA levels are significantly higher in HAM/TSP patients compared with ACs (Yamano et al., 2002). This is in agreement with high levels of serum antibodies to HTLV-1 in HAM/TSP patients (Dekaban et al., 1994). The detection of HTLV-1-specific antibodies in the cerebrospinal fluid and the pX mRNA in the spinal cord were also reported in HAM/TSP patients (Lehky et al., 1995; Gessain, 1996). These observations indicate that HTLV-1 expression is elevated in HAM/TSP patients generally, and also in the spinal cord.

Little is known about the difference in the levels of HTLV-1 expression between tissues in humans. In transgenic mice carrying the pX gene driven by the HTLV-1 long terminal repeat (LTR), RNA expression of the transgene is detectable only in selected organs, including the central nervous system, eyes, salivary glands, and joints (Iwakura et al., 1991). Transgenic mice and rats with the pX gene often develop arthritis and other collagen-vascular inflammatory conditions (Iwakura et al., 1991; Yamazaki et al., 1997). This is partly explained by Tax-mediated activation of NF-κB, a key transcription factor for multiple inflammatory cytokine production. In addition, a certain WKAH strain exhibits HAM/TSP-like symptoms after HTLV-1 infection. This rat model of HAM/TSP shows increased Tax mRNA expression in the spinal cord before the manifestation of the symptoms, suggesting that viral expression in the spinal cord may be the primary event (Tomaru et al., 1996). Reduced IFN-γ production in the spinal cord has been suggested in this particular rat strain (Miyatake et al., 2006).

## DIFFERENT HTLV-1-SPECIFIC T-CELL RESPONSES BETWEEN DISEASES

### ANTI-TUMOR AND ANTI-VIRAL SURVEILLANCE BY HTLV-1-SPECIFIC T-CELLS

The strength of the host T-cell response against HTLV-1 differs among diseases. CD8^+^ HTLV-1-specific CTL responses are activated in HAM/TSP patients but not in ATL patients (Jacobson et al., 1990; Parker et al., 1992; Arnulf et al., 2004; Takamori et al., 2011). These CTLs mainly recognize HTLV-1 Tax and kill HTLV-1-infected cells *in vitro *(Jacobson et al., 1990) (Kannagi et al., 1991). The HTLV-1 envelope protein is also a major target, especially for CD4^+^ CTLs (Goon et al., 2004). Other viral antigens, including polymerase (Elovaara et al., 1993), TOF, ROF (Pique et al., 2000), and HBZ, (Macnamara et al., 2010) have also been shown to be targets of CTLs. Elimination of CD8^+^ cells from the PBMCs from HAM/TSP patients induces HTLV-1 expression during subsequent cell culture (Asquith et al., 2005), clearly indicating that CD8^+^ HTLV-1-specific CTLs contribute to the control of HTLV-1-infected cells.

A series of experiments using a rat model of HTLV-1-infected T-cell lymphoma indicated that inhibition of the T-cell response accelerated tumor development (Hanabuchi et al., 2000), and further showed that vaccination with a Tax-encoding DNA or peptides corresponding to a major epitope for Tax-specific CTLs lead to the eradication of such tumors (Ohashi et al., 2000; Hanabuchi et al., 2001). In a different rat model of HTLV-1 infection, oral HTLV-1 infection induced HTLV-1-specific T-cell tolerance and caused an elevation in the proviral load, while re-immunization of these rats resulted in the recovery of HTLV-1-specific T-cell responses and caused a reduction in the proviral loads (Hasegawa et al., 2003; Komori et al., 2006). Similarly, patients carrying HTLV-1 developed ATL after liver transplantation, when immunosuppressants were administered (Kawano et al., 2006; Suzuki et al., 2006). These findings suggest that HTLV-1-specific T-cells, especially Tax-specific CTLs, play important roles in anti-tumor and anti-viral surveillance in HTLV-1 infection.

The pathological significance of HTLV-1-specific T-cells activated in HAM/TSP patients remains controversial (Jacobson, 1995; Osame, 2002). Activated CTLs produce IFN-γ or TNF-α, which might potentially participate in inflammation in HAM/TSP. However, activation of Tax-specific CTLs could also merely be a result of elevated viral expression in these individuals. HLA-A02-positive individuals in HAM/TSP patients are less frequent compared with the control population, indicating a protective role of HLA-A02 against HAM/TSP. Since HLA-A02 can present a major epitope of HTLV-1 Tax, the strong CTL response induced is thought to mediate the protective effect of HLA-A02 (Jeffery et al., 1999). The association of the protective HLAs with epitopes favoring HBZ-specific CTLs has also been suggested (Macnamara et al., 2010).

### SELECTIVE IMPAIRMENT OF HTLV-1-SPECIFIC T-CELLS IN EARLY STAGES OF ATL, A POTENTIAL RISK FOR ATL

**FIGURE 2:**
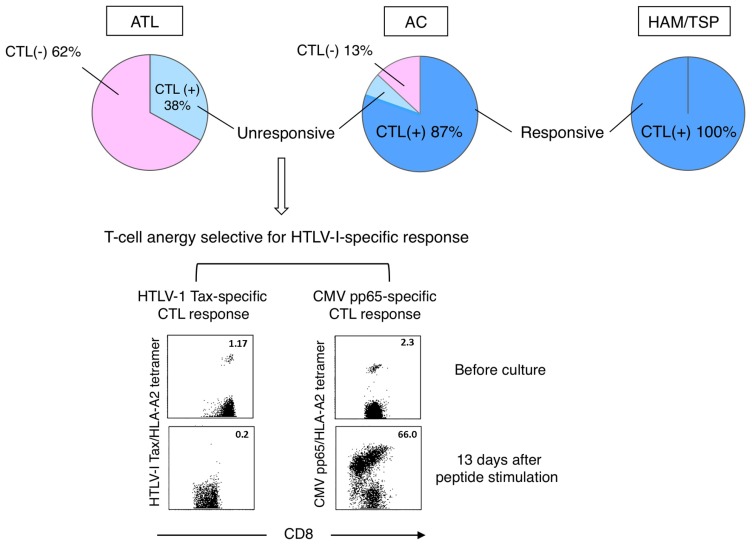
**Selective loss or anergy of Tax-specific CTLs in a subset of ACs.** Incidences of Tax-specific CTL detection in CD8^+^ PBMC vary among diseases (top). Tax-specific CTLs detected in HAM/TSP patients and most of ACs (dark blue) were functional, while those in cATL patients and a subset of ACs (light blue) were anergic to antigen stimulation. The CTL anergy was selective for HTLV-1-specific response (bottom; Takamori et al., 2011).

We previously identified some major epitopes recognized by HTLV-1-specific CTLs presented by HLA-A2, -A11, or -A24 through analysis of CTLs collected from ATL patients after HSCT or collected from HAM/TSP patients (Kannagi et al., 1992; Harashima et al., 2004, 2005).The identification of these epitopes allowed us to monitor HTLV-1-specific CTLs and analyze their functions *ex vivo* by using antigen/HLA tetrameric complexes. In our study using Tax-specific tetramers on HLA-A2, -A11, or -A24-positive individuals, we detected Tax-specific CTLs in 100% of HAM/TSP patients, 87% of ACs, and 38% of chronic ATL (cATL) patients tested (**Figure 2**; Takamori et al., 2011).

It is interesting that Tax-specific CTLs were detectable also in cATL patients, although their frequency among peripheral CD8^+^ cells is low. However, these CTLs were anergic as they neither proliferated nor produced IFN-γ upon peptide stimulation. In contrast, Tax-specific CTLs in HAM/TSP patients were highly active even without stimulation, and their response was further enhanced by stimulation.

Amongst ACs, Tax-specific CTLs are detectable in the majority but not a small subset of individuals. Although Tax-specific CTLs detected in ACs are mostly functional, sporadic AC cases with impaired CTL responses to peptide-stimulation analogous to ATL patients have been identified. Interestingly, such functional impairment of CTLs seems selective to HTLV-1-specific responses, as cytomegalovirus (CMV)-specific CTLs remain functional (**Figure 2**; Takamori et al., 2011). Similar dysfunctions of Tax-specific but not CMV-specific CTLs are found in smoldering ATL, an early stage of ATL without clinically apparent lymphoproliferation. These findings suggest that the scarcity and/or dysfunction of Tax-specific CTLs are not merely the result of ATL, but represent host conditions in a subset of HTLV-1 carriers at asymptomatic stages. A reduced number and/or dysfunction of Tax-specific CTLs could thus represent an underlying risk factor for the development of ATL.

Epidemiological studies indicated that increased numbers of abnormal lymphocytes or HTLV-1 proviral loads are risk factors for the development of ATL (Tajima, 1990; Hisada et al., 1998). However, elevated HTLV-1 proviral loads are also detected in HAM/TSP patients, and do not discriminate risks for ATL and HAM/TSP (Nagai et al., 1998). The immunological studies described above suggested that insufficiency in host T-cell responses against HTLV-1 might be another risk factor for ATL. We therefore suggest that the combination of elevated proviral loads and low HTLV-1-specific T-cell responses may represent a more selective indicator for the risk of develop-ing ATL.

### MECHANISMS OF T-CELL SUPPRESSION IN HTLV-1 INFECTION

It is known that ATL is often associated with severe immune suppression (Tashiro et al., 1992), and a small number of studies reported general immune suppression also in ACs (Imai and Hinuma, 1983). The mechanism of general immune suppression in these individuals is not known. ATL cells are positive for CD4, CD25, CCR4, and frequently express Foxp3, all of which match the phenotype of regulatory T-cells. If ATL cells function as Treg cells, this would be a strong reason for the observed general immune suppression (Karube et al., 2004). There are reports of increased numbers of Foxp3-expressing Treg cells in the HTLV-1-negative cell population in HAM/TSP patients (Toulza et al., 2008). Recent studies reported that HBZ is potentially involved in immune suppression by enhancing TGF-β signaling and suppressing Th1 cytokine production (Zhao et al., 2011; Sugata et al., 2012).

As mentioned above, the insufficient Tax-specific CTL response observed in the early stages of ATL and in a subpopulation of ACs was selective for the response to HTLV-1, and did not affect CMV-targeting CTLs. From this differential effect we deduce that other HTLV-1-specific suppressive mechanisms are active, in addition to the general suppression. In general, antigen-specific T-cell suppression can be induced by immune tolerance and T-cell exhaustion. In HTLV-1 infection, vertical infection could be a reason for T-cell tolerance. In animal models, oral HTLV-1 infection induces T-cell tolerance to HTLV-1, resulting in increased levels of proviral loads (Hasegawa et al., 2003). Since vertical infection of HTLV-1 is established mainly through breast feeding (Kinoshita et al., 1987), it may induce both new-born tolerance and oral tolerance. This might partly explain the epidemiological finding that vertical HTLV-1 infection is one of the risk factors for ATL (Tajima, 1990).

Besides immune tolerance, T-cell suppression can also be caused by T-cell exhaustion, which may be a consequence of continuous expression of HTLV-1 antigens *in vivo*. Expression of PD-1 in Tax-specific CTLs has been reported (Kozako et al., 2009), although the involvement of this molecule in the suppression of CTLs in HTLV-1-infected individuals is still controversial (Takamori et al., 2011). The relevance of other antigens remains unknown, as for example recent studies revealed that Tax-specific CTLs in HAM/TSP patients express reduced levels of Tim3, one of the T-cell exhaustion markers, despite high viral gene expression in these patients (Abdelbary et al., 2011; Ndhlovu et al., 2011).

## IMPACT OF TYPE-I IFNs IN CONTROLLING HTLV-1 EXPRESSION

### INDUCTION OF TYPE-I IFN RESPONSE BY HTLV-1 INFECTION

Various viruses induce type-I IFN responses. In HTLV-1 infection, however, the number of studies investigating a putative HTLV-1-induced type-I IFN response is limited. One of the reasons is that efficient HTLV-1 infection is mediated mainly through cell–cell contact. A recent report indicated that addition of cell-free HTLV-1 particles propagated using an HTLV-1 molecular clone to plasmacytoid dendritic cells (pDCs) induced a type-I IFN response through Toll-like receptor 7 (TLR7; Colisson et al., 2010). pDCs are a major producer of type-I IFNs, and are reported to be susceptible to HTLV-1 infection (Hishizawa et al., 2004; Jones et al., 2008). In ATL patients, the number of pDCs is decreased, and the remaining pDCs lack the ability to produce IFN-α (Hishizawa et al., 2004).

At cell–cell contacts between HTLV-1-infected T-cells and stromal cells, we found that HTLV-1 induced a type-I IFN response in the stromal cells, suggesting an involvement of pattern recognition molecules other than TLR7. However, the precise mechanisms of HTLV-1-induced type-I IFN responses remain to be clarified.

### SUPPRESSION OF HTLV-1 EXPRESSION BY TYPE-I IFNs

**FIGURE 3:**
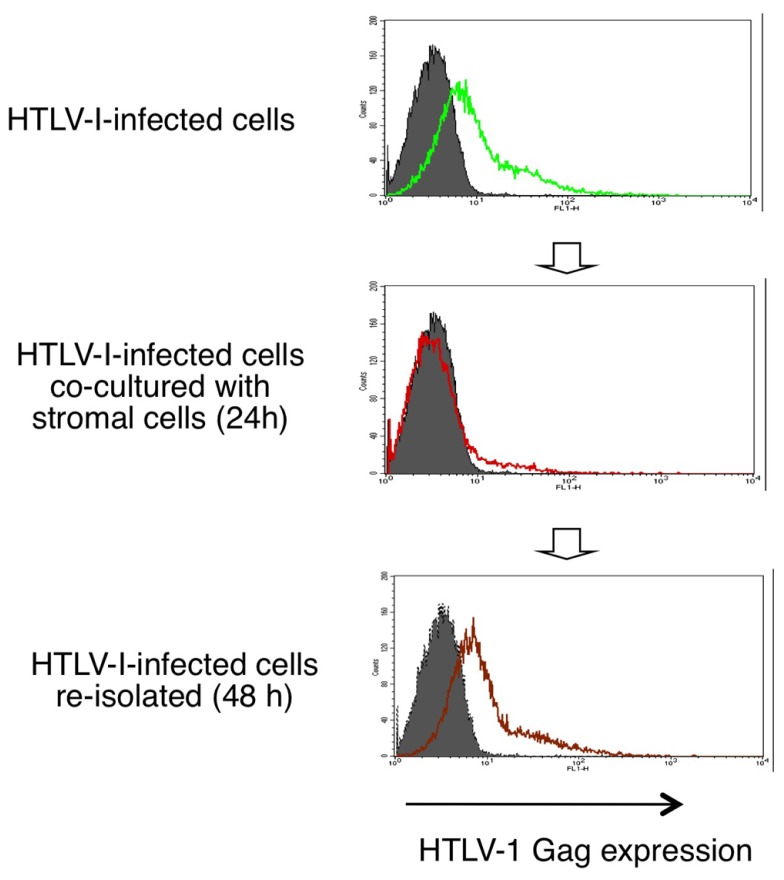
**Reversible suppression of HTLV-1 expression by stromal cells through type-I IFNs.** HTLV-1 protein expression in HTLV-1-infected T-cells are markedly decreased when co-cultured with stromal cells and recovers after re-isolation from the co-cultures (Kinpara et al., 2009).

HTLV-1 mRNA and protein expression in HTLV-1-infected T-cells are markedly decreased when co-cultured with stromal cells such as epithelial cells and fibroblasts. This suppression of HTLV-1 expression is inhibited by blocking the IFN-α/β receptor, and is therefore thought to be mediated through type-I IFN responses (**Figure 3**; Kinpara et al., 2009).

Interestingly, when infected cells were re-isolated from the co-cultures, viral expression was restored to the original level over the following 48 h (**Figure 3**). This phenomenon resembles the induction of HTLV-1 expression in freshly isolated ATL cells after culture *in vitro*. Type-I IFNs might therefore explain the long-puzzling observation that HTLV-1 expression is suppressed *in vivo*. In support of this notion, viral expression in HTLV-1-infected cells was significantly suppressed when injected into wild-type mice but not into IFN regulatory factor-7-knockout mice, which are deficient in most type-I IFN responses (Kinpara et al., 2009).

In general, type-I IFNs suppress viral replication mostly at post-transcriptional level. Since HTLV-1 transcription is regulated by transactivation of its own LTR, mainly through cyclic AMP (cAMP) response element-like repeats by the Tax protein (Fujisawa et al., 1985; Sodroski et al., 1985), limitation of this protein below a certain level will efficiently reduce HTLV-1 expression to a basal level. Involvement of inducible cAMP early repressor (ICER) and transducer of regulated CREB protein 2 (TORC2) in the inhibition of HTLV-1 transactivation has also been suggested (Newbound et al., 2000; Jiang et al., 2009).

Addition of IFNs alone also elicits suppressive effects in HTLV-1 expression. However, the levels of suppressive effects differ among studies. In HTLV-1-infected cell lines, it has been reported that IFN-α2a decreased HTLV-1 assembly and viral release but not viral protein synthesis (Feng et al., 2003).

### RESISTANCE OF HTLV-1 AGAINST TYPE-I IFN SIGNALING

As is the case with many other viruses, HTLV-1 has developed strategies to evade IFN responses. It has been reported that HTLV-1 infection reduces the phosphorylation of tyrosine kinase 2 (TYK2) and signal transducer and transcriptional activator 2 (STAT2; Feng and Ratner, 2008), and that Tax inhibit the induction of IFN-stimulated genes (ISGs) by competing with CREB binding protein/p300 (Zhang et al., 2008). Recent reports also suggest that Tax-mediated up-regulation of suppressor of cytokine signaling 1 (SOCS1) inhibits IFN signaling (Oliere et al., 2010; Charoenthongtrakul et al., 2011). However, expression levels of Tax protein are low *in vivo*, and it is unclear to what extent the evading mechanisms observed *in vitro* are effective *in vivo*.

It has been reported that a combination therapy of AZT and IFN-α is effective for the treatment of ATL (Hermine et al., 1995), indicating that HTLV-1-infected cells retain some susceptibility to IFNs *in vivo*. Intriguingly, this combination of AZT/IFN-α does not affect HTLV-1-infected cells *in vitro *(Bazarbachi et al., 2000), and the mechanistic effect of this therapy is not known. The discrepancy in the therapeutic effects *in vivo* and *in vitro* is presumably due to the different status of HTLV-1-infected cells in the two systems.

AZT/IFN-α is not a radical therapy, and ATL relapses are frequently observed after cessation of the therapy (Hermine et al., 2002), suggesting that AZT/IFN-α may not be cytocidal but rather has static effects on infected cells. Another combination therapy of arsenic trioxide and IFN-α shows more favorable therapeutic effects *in vivo*, and also shows proteolysis of Tax in HTLV-1-infected cells *in vitro* (El Hajj et al., 2010). IFN-α or β alone appears less effective for the treatment of HAM/TSP, but does show some therapeutic effects, especially during the early stages of HAM/TSP (Izumo et al., 1996; Saito et al., 2004).

### IFN RESPONSES IN HTLV-1-INFECTED INDIVIDUALS

A recent study revealed up-regulation of SOCS1 in CD4^+^ cells of HAM/TSP patients, which caused enhanced viral expression through inhibition of type-I IFN signaling (Oliere et al., 2010). At the same time, a different study showed that HTLV-1 Tax up-regulates SOCS1 (Charoenthongtrakul et al., 2011). These findings indicate that up-regulation of SOCS1 might be a result and/or cause of enhanced viral expression in HAM/TSP. Another recent study using gene expression array analysis reported up-regulation of a subset of ISGs, including STAT1, CD64, FAS, and CXCL10, especially in the neutrophil and monocyte fractions from peripheral blood of HAM/TSP patients (Tattermusch et al., 2012). This suggests that type-I IFN responses were induced in these cell populations directly or indirectly by HTLV-1, although type-I IFN production in these cells was not clear. The strong HTLV-1-specific T-cell response in these patients might also cause such effects through IFN-γ production.

The signature of IFN responses in the peripheral blood of HAM/TSP patients left an unanswered question what enhances the basal level of viral expression in these patients. Increased inflammatory cytokines in HAM/TSP patients might be candidates to enhance viral expression, but again these could be a result and/or cause of enhanced HTLV-1 expression.

## THE RELATIONSHIP AMONG VIRAL EXPRESSION, HOST IMMUNITY, AND VIRAL PATHOGENESIS

It is speculated that the status of viral expression and host immunity may differ among various tissues *in vivo*. Therefore, it is difficult to estimate HTLV-1 status in the entire body based on the information gained only from peripheral blood. Nevertheless, the recent findings about innate immunity described above provide clues as to how the current knowledge around HTLV-1 expression and host immunity can be integrated, especially when they so closely interact and have both causes and effects on each other.

Type-I IFNs are likely to be the representative factor to control HTLV-1 expression, and HTLV-1-specific T-cells survey infected cells to limit their growth. The suppression of viral expression might interfere with the efficacy of HTLV-1-specific T-cells by reducing the levels of target molecules, even in hosts with a functional HTLV-1-specific T-cell response. The resulting low efficiency of T-cell surveillance would be one of the mechanisms behind persistent HTLV-1 infection, although it seems that T-cells would still contribute to the control of HTLV-1-infected cell growth to some extent.

Despite the negative impact on T-cell surveillance, the suppression of viral expression is important for the host to reduce viral pathogenesis since Tax has a strong ability to activate NF-κB, which is critical for the induction of inflammation or cell growth signaling.

**FIGURE 4:**
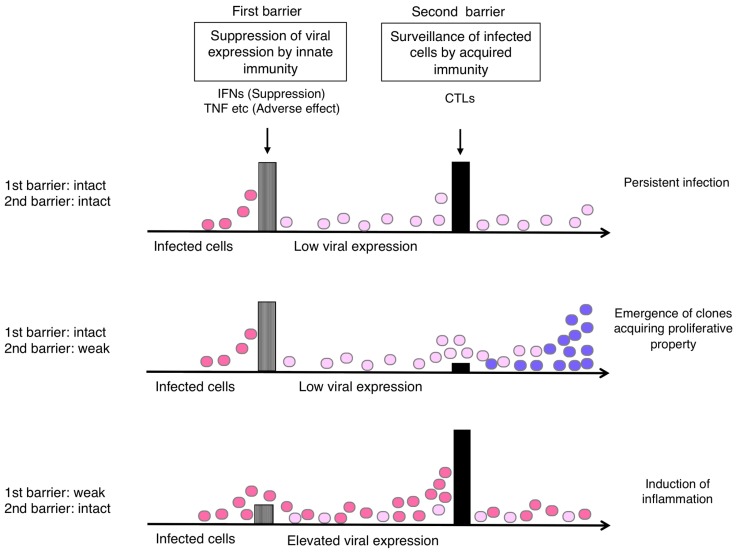
**Hypothetical relationship of the host immune balance to the status of HTLV-1-infected cells and viral pathogenesis.** If viral expression is well controlled (barrier 1: intact), the viral pathogenesis may not be apparent until malignant cell clones appear through T-cell surveillance (barrier 2). If the T-cell defense is insufficient (barrier 2: weak), the emergence of such clones may occur earlier. If the viral expression is not well controlled (barrier 1: weak), virus-induced inflammation may become apparent, but it also activates HTLV-1-specific T-cells that limit further growth of infected cells.

Supposing that the suppression of viral expression is the first barrier and T-cell surveillance of infected cells is the second barrier in the host defense, the balance of these barriers would influence the status of HTLV-1-infected cells *in vivo*. A conceivable scenario is as follows (**Figure 4**).

If viral expression is well controlled, the viral pathogenesis may not be apparent until malignant cell clones appear through the process of clonal evolution in the infected cell reservoir. This might explain the long incubation time for ATL development. In the absence of effective T-cell responses, the emergence of such clones may occur earlier, as clonal survival may be more likely.

In contrast, if the suppression of viral expression is insufficient, either by insufficient IFN response or increased inflammatory cytokines, viral pathogenesis will become apparent and symptoms will be exhibited, especially in the tissues where viral proteins reach functional levels. In this case, however, the elevated levels of viral expression would also activate HTLV-1-specific T-cells, which potentially limit further growth of infected cells.

## CONCLUSION

The status of HTLV-1-specific T-cell response has been shown to be a determinant of HTLV-1-mediated diseases because of its anti-tumor and anti-viral effects. The selective impairment of HTLV-1-specific T-cell responses in early stages of ATL patients implies the presence of HTLV-1-specific suppressive mechanisms. The combination of insufficient HTLV-1-specific T-cell response and elevated proviral load may allow the identification of a group with a high risk for the development of ATL. In addition, vaccines that augment HTLV-1-specific T-cell responses may prove beneficial in reducing the risk in such a subpopu-lation.

The status of HTLV-1 expression can be another determinant of HTLV-1-mediated diseases. Suppression of viral expression contributes to reduced viral pathogenesis, although it may, at the same time, partially interfere with T-cell surveillance. Host innate immunity, especially type-I IFN, is a candidate for the regulation of viral expression.

Thus, both acquired and innate immunity can be host determinants that modulate HTLV-1-associated diseases. The involvement of the two control systems and their partially conflicting effects on one another may explain why the same virus can cause different diseases after a long incubation time. Further studies will elucidate the precise mechanisms for the regulation of host immunity and viral expression, and thereby provide insights for the prediction of disease risks, as well as new targets for the prevention of HTLV-1-mediated diseases.

## Conflict of Interest Statement

The authors declare that the research was conducted in the absence of any commercial or financial relationships that could be construed as a potential conflict of interest.

## References

[B1] Abdelbary N. H. Abdullah H. M. Matsuzaki T. Hayashi D. Tanaka Y. Takashima H. Izumo S. Kubota R. (2011). Reduced Tim-3 expression on human T-lymphotropic virus type I (HTLV-I) Tax-specific cytotoxic T lymphocytes in HTLV-I infection. *J. Infect. Dis.* 203 948–959 21402546 10.1093/infdis/jiq153

[B2] Arnulf B. Thorel M. Poirot Y. Tamouza R. Boulanger E. Jaccard A. Oksenhendler E. Hermine O. Pique C. (2004). Loss of the *ex vivo* but not the reinducible CD8+ T-cell response to Tax in human T-cell leukemia virus type 1-infected patients with adult T-cell leukemia/lymphoma. *Leukemia* 18 126–132 14574331 10.1038/sj.leu.2403176

[B3] Asquith B. Mosley A. J. Barfield A. Marshall S. E. Heaps A. Goon P. Hanon E. Tanaka Y. Taylor G. P. Bangham C. R. (2005). A functional CD8+ cell assay reveals individual variation in CD8+ cell antiviral efficacy and explains differences in human T-lymphotropic virus type 1 proviral load. *J. Gen. Virol.* 86 1515–1523 15831965 10.1099/vir.0.80766-0

[B4] Bangham C. R. Osame M. (2005). Cellular immune response to HTLV-1. *Oncogene* 24 6035–6046 16155610 10.1038/sj.onc.1208970

[B5] Bazarbachi A. Nasr R. El-Sabban M. E. Mahe A. Mahieux R. Gessain A. Darwiche N. Dbaibo G. Kersual J. Zermati Y. Dianoux L. Chelbi-Alix M. K. De The H. Hermine O. (2000). Evidence against a direct cytotoxic effect of alpha interferon and zidovudine in HTLV-I associated adult T cell leukemia/lymphoma. *Leukemia* 14 716–721 10764160 10.1038/sj.leu.2401742

[B6] Charoenthongtrakul S. Zhou Q. Shembade N. Harhaj N. S. Harhaj E. W. (2011). Human T cell leukemia virus type 1 Tax inhibits innate antiviral signaling via NF-kappaB-dependent induction of SOCS1. *J. Virol.* 85 6955–6962 21593151 10.1128/JVI.00007-11 PMC3126571

[B7] Colisson R. Barblu L. Gras C. Raynaud F. Hadj-Slimane R. Pique C. Hermine O. Lepelletier Y. Herbeuval J. P. (2010). Free HTLV-1 induces TLR7-dependent innate immune response and TRAIL relocalization in killer plasmacytoid dendritic cells. *Blood* 115 2177–2185 20007807 10.1182/blood-2009-06-224741

[B8] Daenke S. Nightingale S. Cruickshank J. K. Bangham C. R. (1990). Sequence variants of human T-cell lymphotropic virus type I from patients with tropical spastic paraparesis and adult T-cell leukemia do not distinguish neurological from leukemic isolates. *J. Virol.* 64 1278–1282 2304144 10.1128/jvi.64.3.1278-1282.1990 PMC249244

[B9] Dekaban G. A. King E. E. Arp J. Palker T. J. Rice G. P. (1994). Comparative analysis of the antibody response to the HTLV-I gag and env proteins in HTLV-I asymptomatic carriers and HAM/TSP patients: an isotype and subclass analysis. *Scand. J. Immunol.* 40 171–180 8047838 10.1111/j.1365-3083.1994.tb03447.x

[B10] El Hajj H. El-Sabban M. Hasegawa H. Zaatari G. Ablain J. Saab S. T. Janin A. Mahfouz R. Nasr R. Kfoury Y. Nicot C. Hermine O. Hall W. De The H. Bazarbachi A. (2010). Therapy-induced selective loss of leukemia-initiating activity in murine adult T cell leukemia. *J. Exp. Med.* 207 2785–2792 21135137 10.1084/jem.20101095 PMC3005222

[B11] Elovaara I. Koenig S. Brewah A. Y. Woods R. M. Lehky T. Jacobson S. (1993). High human T cell lymphotropic virus type 1 (HTLV-1)-specific precursor cytotoxic T lymphocyte frequencies in patients with HTLV-1-associated neurological disease. *J. Exp. Med.* 177 1567–1573 8496677 10.1084/jem.177.6.1567 PMC2191033

[B12] Feng X. Heyden N. V. Ratner L. (2003). Alpha interferon inhibits human T-cell leukemia virus type 1 assembly by preventing Gag interaction with rafts. *J. Virol.* 77 13389–13395 14645593 10.1128/JVI.77.24.13389-13395.2003 PMC296084

[B13] Feng X. Ratner L. (2008). Human T-cell leukemia virus type 1 blunts signaling by interferon alpha. *Virology* 374 210–216 18234266 10.1016/j.virol.2007.12.036 PMC2373983

[B14] Fujisawa J. Seiki M. Kiyokawa T. Yoshida M. (1985). Functional activation of the long terminal repeat of human T-cell leukemia virus type I by a trans-acting factor. *Proc. Natl. Acad. Sci. U.S.A.* 82 2277–2281 2986109 10.1073/pnas.82.8.2277 PMC397540

[B15] Furukawa Y. Osame M. Kubota R. Tara M. Yoshida M. (1995). Human T-cell leukemia virus type-1 (HTLV-1) Tax is expressed at the same level in infected cells of HTLV-1-associated myelopathy or tropical spastic paraparesis patients as in asymptomatic carriers but at a lower level in adult T-cell leukemia cells. *Blood* 85 1865–1870 7703492

[B16] Gessain A. (1996). Virological aspects of tropical spastic parapare-sis/HTLV-I associated myelopathy and HTLV-I infection. *J. Neurovirol.* 2 299–306 8912206 10.3109/13550289609146894

[B17] Gessain A. Barin F. Vernant J. C. Gout O. Maurs L. Calender A De The G. (1985). Antibodies to human T-lymphotropic virus type-I in patients with tropical spastic paraparesis. *Lancet* 2 407–410 2863442 10.1016/s0140-6736(85)92734-5

[B18] Goon P. K. Igakura T. Hanon E. Mosley A. J. Barfield A. Barnard A. L. Kaftantzi L. Tanaka Y. Taylor G. P. Weber J. N. Bangham C. R. (2004). Human T cell lymphotropic virus type I (HTLV-I)-specific CD4+ T cells: immunodominance hierarchy and preferential infection with HTLV-I. *J. Immunol.* 172 1735–1743 14734756 10.4049/jimmunol.172.3.1735

[B19] Grassmann R. Aboud M. Jeang K. T. (2005). Molecular mechanisms of cellular transformation by HTLV-1 Tax. *Oncogene* 24 5976–5985 16155604 10.1038/sj.onc.1208978

[B20] Hanabuchi S. Ohashi T. Koya Y. Kato H. Hasegawa A. Takemura F. Masuda T. Kannagi M. (2001). Regression of human T-cell leukemia virus type I (HTLV-I)-associated lymphomas in a rat model: peptide-induced T-cell immunity. *J. Natl. Cancer Inst.* 93 1775–1783 11734593 10.1093/jnci/93.23.1775

[B21] Hanabuchi S. Ohashi T. Koya Y. Kato H. Takemura F. Hirokawa K. Yoshiki T. Yagita H. Okumura K. Kannagi M. (2000). Development of human T-cell leukemia virus type 1-transformed tumors in rats following suppression of T-cell immunity by CD80 and CD86 blockade. *J. Virol.* 74 428–435 10590132 10.1128/jvi.74.1.428-435.2000 PMC111554

[B22] Hanon E. Hall S. Taylor G. P. Saito M. Davis R. Tanaka Y. Usuku K. Osame M. Weber J. N. Bangham C. R. (2000). Abundant tax protein expression in CD4+ T cells infected with human T-cell lymphotropic virus type I (HTLV-I) is prevented by cytotoxic T lymphocytes. *Blood* 95 1386–1392 10666215

[B23] Harashima N. Kurihara K. Utsunomiya A. Tanosaki R. Hanabuchi S. Masuda M. Ohashi T. Fukui F. Hasegawa A. Masuda T. Takaue Y. Okamura J. Kannagi M. (2004). Graft-versus-Tax response in adult T-cell leukemia patients after hematopoietic stem cell transplantation. *Cancer Res.* 64 391–399 14729650 10.1158/0008-5472.can-03-1452

[B24] Harashima N. Tanosaki R. Shimizu Y. Kurihara K. Masuda T. Okamura J. Kannagi M. (2005). Identification of two new HLA-A*1101-restricted tax epitopes recognized by cytotoxic T lymphocytes in an adult T-cell leukemia patient after hematopoietic stem cell transplantation. *J. Virol.* 79 10088–10092 16014972 10.1128/JVI.79.15.10088-10092.2005 PMC1181560

[B25] Hasegawa A. Ohashi T. Hanabuchi S. Kato H. Takemura F. Masuda T. Kannagi M. (2003). Expansion of human T-cell leukemia virus type 1 (HTLV-1) reservoir in orally infected rats: inverse correlation with HTLV-1-specific cellular immune response. *J. Virol.* 77 2956–2963 12584320 10.1128/JVI.77.5.2956-2963.2003 PMC149753

[B26] Hermine O. Allard I. Levy V. Arnulf B. Gessain A. Bazarbachi A. (2002). A prospective phase II clinical trial with the use of zidovudine and interferon-alpha in the acute and lymphoma forms of adult T-cell leukemia/lymphoma. *Hematol. J.* 3 276–282 12522449 10.1038/sj.thj.6200195

[B27] Hermine O. Bouscary D. Gessain A. Turlure P. Leblond V. Franck N. Buzyn-Veil A. Rio B. Macintyre E. Dreyfus F. Bazarbachi A. (1995). Brief report: treatment of adult T-cell leukemia-lymphoma with zidovudine and interferon alfa. *N. Engl. J. Med.* 332 1749–1751 7760891 10.1056/NEJM199506293322604

[B28] Hilburn S. Rowan A. Demontis M. A. Macnamara A. Asquith B. Bangham C. R. Taylor G. P. (2011). *In vivo* expression of human T-lymphotropic virus type 1 basic leucine-zipper protein generates specific CD8+ and CD4+ T-lymphocyte responses that correlate with clinical outcome. *J. Infect. Dis.* 203 529–536 21208912 10.1093/infdis/jiq078 PMC3071236

[B29] Hinuma Y. Gotoh Y. Sugamura K. Nagata K. Goto T. Nakai M. Kamada N. Matsumoto T. Kinoshita K. (1982). A retrovirus associated with human adult T-cell leukemia: *in vitro* activation. *Gann* 73 341–344 6981536

[B30] Hinuma Y. Nagata K. Hanaoka M. Nakai M. Matsumoto T. Kinoshita K. I. Shirakawa S. Miyoshi I. (1981). Adult T-cell leukemia: antigen in an ATL cell line and detection of antibodies to the antigen in human sera. *Proc. Natl. Acad. Sci. U.S.A.* 78 6476–6480 7031654 10.1073/pnas.78.10.6476 PMC349062

[B31] Hisada M. Okayama A. Tachibana N. Stuver S. O. Spiegelman D. L. Tsubouchi H. Mueller N. E. (1998). Predictors of level of circulating abnormal lymphocytes among human T-lymphotropic virus type I carriers in Japan. *Int. J. Cancer* 77 188–192 9650550 10.1002/(sici)1097-0215(19980717)77:2<188::aid-ijc3>3.0.co;2-m

[B32] Hishizawa M. Imada K. Kitawaki T. Ueda M. Kadowaki N. Uchiyama T. (2004). Depletion and impaired interferon-alpha-producing capacity of blood plasmacytoid dendritic cells in human T-cell leukaemia virus type I-infected individuals. *Br. J. Haematol.* 125 568–575 15147371 10.1111/j.1365-2141.2004.04956.x

[B33] Imai J. Hinuma Y. (1983). Epstein–Barr virus-specific antibodies in patients with adult T-cell leukemia (ATL) and healthy ATL virus-carriers. *Int. J. Cancer* 31 197–200 6298126 10.1002/ijc.2910310210

[B34] Iwakura Y. Tosu M. Yoshida E. Takiguchi M. Sato K. Kitajima I. Nishioka K. Yamamoto K. Takeda T. Hatanaka M. Yamamoto H. Sekiguchi T. (1991). Induction of inflammatory arthropathy resembling rheumatoid arthritis in mice transgenic for HTLV-I. *Science* 253 1026–1028 1887217 10.1126/science.1887217

[B35] Izumo S. Goto I. Itoyama Y. Okajima T. Watanabe S. Kuroda Y. Araki S. Mori M. Nagataki S. Matsukura S. Akamine T. Nakagawa M. Yamamoto I. Osame M. (1996). Interferon-alpha is effective in HTLV-I-associated myelopathy: a multicenter, randomized, double-blind, controlled trial. *Neurology* 46 1016–1021 8780082 10.1212/wnl.46.4.1016

[B36] Jacobson S. (1995). Human T lymphotropic virus, type-I myelopathy: an immunopathologically mediated chronic progressive disease of the central nervous system. *Curr. Opin. Neurol.* 8 179–183 7551116

[B37] Jacobson S. Shida H. Mcfarlin D. E. Fauci A. S. Koenig S. (1990). Circulating CD8+ cytotoxic T lymphocytes specific for HTLV-I pX in patients with HTLV-I associated neurological disease. *Nature* 348 245–248 2146511 10.1038/348245a0

[B38] Jeang K. T. Giam C. Z. Majone F. Aboud M. (2004). Life, death, and tax: role of HTLV-I oncoprotein in genetic instability and cellular transformation. *J. Biol. Chem.* 279 31991–31994 15090550 10.1074/jbc.R400009200

[B39] Jeffery K. J. Usuku K. Hall S. E. Matsumoto W. Taylor G. P. Procter J. Bunce M. Ogg G. S. Welsh K. I. Weber J. N. Lloyd A. L. Nowak M. A. Nagai M. Kodama D. Izumo S. Osame M. Bangham C. R. (1999). HLA alleles determine human T-lymphotropic virus-I (HTLV-I) proviral load and the risk of HTLV-I-associated myelopathy. *Proc. Natl. Acad. Sci. U.S.A.* 96 3848–3853 10097126 10.1073/pnas.96.7.3848 PMC22383

[B40] Jiang S. Inada T. Tanaka M. Furuta R. A. Shingu K. Fujisawa J. (2009). Involvement of TORC2, a CREB co-activator, in the *in vivo*-specific transcriptional control of HTLV-1. *Retrovirology* 6 73 10.1186/1742-4690-6-73 PMC2734550 19664292

[B41] Jones K. S. Petrow-Sadowski C. Huang Y. K. Bertolette D. C. Ruscetti F. W. (2008). Cell-free HTLV-1 infects dendritic cells leading to transmission and transformation of CD4(+) T cells. *Nat. Med.* 14 429–436 18376405 10.1038/nm1745

[B42] Kannagi M. Harada S. Maruyama I. Inoko H. Igarashi H. Kuwashima G. Sato S. Morita M. Kidokoro M. Sugimoto M. Funahashi M. Osame M. Shida H. (1991). Predominant recognition of human T cell leukemia virus type I (HTLV-I) pX gene products by human CD8+ cytotoxic T cells directed against HTLV-I-infected cells. *Int. Immunol.* 3 761–767 1911545 10.1093/intimm/3.8.761

[B43] Kannagi M. Harashima N. Kurihara K. Ohashi T. Utsunomiya A. Tanosaki R. Masuda M. Tomonaga M. Okamura J. (2005). Tumor immunity against adult T-cell leukemia. *Cancer Sci.* 96 249–255 15904464 10.1111/j.1349-7006.2005.00050.x PMC11158966

[B44] Kannagi M. Shida H. Igarashi H. Kuruma K. Murai H. Aono Y. Maruyama I. Osame M. Hattori T. Inoko H. Harasa S. (1992). Target epitope in the Tax protein of human T-cell leukemia virus type I recognized by class I major histocompatibility complex-restricted cytotoxic T cells. *J. Virol.* 66 2928–2933 1373197 10.1128/jvi.66.5.2928-2933.1992 PMC241051

[B45] Karube K. Ohshima K. Tsuchiya T. Yamaguchi T. Kawano R. Suzumiya J. Utsunomiya A. Harada M. Kikuchi M. (2004). Expression of FoxP3, a key molecule in CD4CD25 regulatory T cells, in adult T-cell leukaemia/lymphoma cells. *Br. J. Haematol.* 126 81–84 15198736 10.1111/j.1365-2141.2004.04999.x

[B46] Kawano N. Shimoda K. Ishikawa F. Taketomi A. Yoshizumi T. Shimoda S. Yoshida S. Uozumi K. Suzuki S. Maehara Y. Harada M. (2006). Adult T-cell leukemia development from a human T-cell leukemia virus type I carrier after a living-donor liver transplantation. *Transplantation* 82 840–843 17006333 10.1097/01.tp.0000235186.30113.c7

[B47] Kinoshita K. Amagasaki T. Hino S. Doi H. Yamanouchi K. Ban N. Momita S. Ikeda S. Kamihira S. Ichimaru M. Katamine S. Miyamoto T. Tsuji Y. Ishimaru T. Yamabe T. Ito M. Kamura S. Tsuda T. (1987). Milk-borne transmission of HTLV-I from carrier mothers to their children. *Jpn. J. Cancer Res.* 78 674–680 2887539

[B48] Kinoshita T. Shimoyama M. Tobinai K. Ito M. Ito S. Ikeda S. Tajima K. Shimotohno K. Sugimura T. (1989). Detection of mRNA for the tax1/rex1 gene of human T-cell leukemia virus type I in fresh peripheral blood mononuclear cells of adult T-cell leukemia patients and viral carriers by using the polymerase chain reaction. *Proc. Natl. Acad. Sci. U.S.A.* 86 5620–5624 2787512 10.1073/pnas.86.14.5620 PMC297674

[B49] Kinoshita T. Tsujimoto A. Shimotohno K. (1991). Sequence variations in LTR and env regions of HTLV-I do not discriminate between the virus from patients with HTLV-I-associated myelopathy and adult T-cell leukemia. *Int. J. Cancer* 47 491–495 1995478 10.1002/ijc.2910470403

[B50] Kinpara S. Hasegawa A. Utsunomiya A. Nishitsuji H. Furukawa H. Masuda T. Kannagi M. (2009). Stromal cell-mediated suppression of human T-cell leukemia virus type 1 expression *in vitro* and *in vivo* by type I interferon. *J. Virol.* 83 5101–5108 19264779 10.1128/JVI.02564-08 PMC2682107

[B51] Komori K. Hasegawa A. Kurihara K. Honda T. Yokozeki H. Masuda T. Kannagi M. (2006). Reduction of human T-cell leukemia virus type 1 (HTLV-1) proviral loads in rats orally infected with HTLV-1 by reimmunization with HTLV-1-infected cells. *J. Virol.* 80 7375–7381 16840318 10.1128/JVI.00230-06 PMC1563733

[B52] Kozako T. Yoshimitsu M. Fujiwara H. Masamoto I. Horai S. White Y. Akimoto M. Suzuki S. Matsushita K. Uozumi K. Tei C. Arima N. (2009). PD-1/PD-L1 expression in human T-cell leukemia virus type 1 carriers and adult T-cell leukemia/lymphoma patients. *Leukemia* 23 375–382 18830259 10.1038/leu.2008.272

[B53] Kurihara K. Harashima N. Hanabuchi S. Masuda M. Utsunomiya A. Tanosaki R. Tomonaga M. Ohashi T. Hasegawa A. Masuda T. Okamura J. Tanaka Y. Kannagi M. (2005). Potential immunogenicity of adult T cell leukemia cells *in vivo*. *Int. J. Cancer* 114 257–267 15551352 10.1002/ijc.20737

[B54] Lehky T. J. Fox C. H. Koenig S. Levin M. C. Flerlage N. Izumo S. Sato E. Raine C. S. Osame M. Jacobson S. (1995). Detection of human T-lymphotropic virus type I (HTLV-I) tax RNA in the central nervous system of HTLV-I-associated myelopathy/tropical spastic paraparesis patients by in situ hybridization. *Ann. Neurol.* 37 167–175 7847858 10.1002/ana.410370206

[B55] Macnamara A. Rowan A. Hilburn S. Kadolsky U. Fujiwara H. Suemori K. Yasukawa M. Taylor G. Bangham C. R. Asquith B. (2010). HLA class I binding of HBZ determines outcome in HTLV-1 infection. *PLoS Pathog.* 6 e1001117 10.1371/journal.ppat.1001117 PMC2944806 20886101

[B56] Miyatake Y. Ikeda H. Ishizu A. Baba T. Ichihashi T. Suzuki A. Tomaru U. Kasahara M. Yoshiki T. (2006). Role of neuronal interferon-gamma in the development of myelopathy in rats infected with human T-cell leukemia virus type 1. *Am. J. Pathol.* 169 189–199 16816372 10.2353/ajpath.2006.051225 PMC1698768

[B57] Nagai M. Usuku K. Matsumoto W. Kodama D. Takenouchi N. Moritoyo T. Hashiguchi S. Ichinose M. Bangham C. R. Izumo S. Osame M. (1998). Analysis of HTLV-I proviral load in 202 HAM/TSP patients and 243 asymptomatic HTLV-I carriers: high proviral load strongly predisposes to HAM/TSP. *J. Neurovirol.* 4 586–593 10065900 10.3109/13550289809114225

[B58] Ndhlovu L. C. Leal F. E. Hasenkrug A. M. Jha A. R. Carvalho K. I. Eccles-James I. G. Bruno F. R. Vieira R. G. York V. A. Chew G. M. Jones R. B. Tanaka Y. Neto W. K. Sanabani S. S. Ostrowski M. A. Segurado A. C. Nixon D. F. Kallas E. G. (2011). HTLV-1 tax specific CD8 T cells express low levels of Tim-3 in HTLV-1 infection: implications for progression to neurological complications. *PLoS Negl. Trop. Dis.* 5 e1030 10.1371/journal.pntd.0001030 PMC3082508 21541358

[B59] Newbound G. C. O’Rourke J. P. Collins N. D. Andrews J. M. Dewille J. Lairmore M. D. (2000). Repression of tax-mediated human t-lymphotropic virus type 1 transcription by inducible cAMP early repressor (ICER) protein in peripheral blood mononuclear cells. *J. Med. Virol.* 62 286–292 11002260

[B60] Ohashi T. Hanabuchi S. Kato H. Tateno H. Takemura F. Tsukahara T. Koya Y. Hasegawa A. Masuda T. Kannagi M. (2000). Prevention of adult T-cell leukemia-like lymphoproliferative disease in rats by adoptively transferred T cells from a donor immunized with human T-cell leukemia virus type 1 Tax-coding DNA vaccine. *J. Virol.* 74 9610–9616 11000233 10.1128/jvi.74.20.9610-9616.2000 PMC112393

[B61] Oliere S. Hernandez E. Lezin A. Arguello M. Douville R. Nguyen T. L. Olindo S. Panelatti G. Kazanji M. Wilkinson P. Sekaly R. P. Cesaire R. Hiscott J. (2010). HTLV-1 evades type I interferon antiviral signaling by inducing the suppressor of cytokine signaling 1 (SOCS1). *PLoS Pathog.* 6 e1001177 10.1371/journal.ppat.1001177 PMC2973829 21079688

[B62] Osame M. (2002). Pathological mechanisms of human T-cell lymphotropic virus type I-associated myelopathy (HAM/TSP). *J. Neurovirol.* 8 359–364 12402162 10.1080/13550280260422668

[B63] Osame M. Usuku K. Izumo S. Ijichi N. Amitani H. Igata A. Matsumoto M. Tara M. (1986). HTLV-I associated myelopathy, a new clinical entity. *Lancet* 1 1031–1032 2871307 10.1016/s0140-6736(86)91298-5

[B64] Ozden S. Cochet M. Mikol J. Teixeira A. Gessain A. Pique C. (2004). Direct evidence for a chronic CD8 T-cell-mediated immune reaction to tax within the muscle of a human T-cell leukemia/lymphoma virus type 1-infected patient with sporadic inclusion body myositis. *J. Virol.* 78 10320–10327 15367598 10.1128/JVI.78.19.10320-10327.2004 PMC516372

[B65] Parker C. E. Daenke S. Nightingale S. Bangham C. R. (1992). Activated, HTLV-1-specific cytotoxic T-lymphocytes are found in healthy seropositives as well as in patients with tropical spastic paraparesis. *Virology* 188 628–636 1374983 10.1016/0042-6822(92)90517-s

[B66] Pique C. Ureta-Vidal A. Gessain A. Chancerel B. Gout O. Tamouza R. Agis F. Dokhelar M. C. (2000). Evidence for the chronic *in vivo* production of human T cell leukemia virus type I Rof and Tof proteins from cytotoxic T lymphocytes directed against viral peptides. *J. Exp. Med.* 191 567–572 10662802 10.1084/jem.191.3.567 PMC2195825

[B67] Rende F. Cavallari I. Corradin A. Silic-Benussi M. Toulza F. Toffolo G. M. Tanaka Y. Jacobson S. Taylor G. P. D’Agostino D. M. Bangham C. R. Ciminale V. (2011). Kinetics and intracellular compartmentalization of HTLV-1 gene expression: nuclear retention of HBZ mRNAs. *Blood* 117 4855–4859 21398577 10.1182/blood-2010-11-316463 PMC5292588

[B68] Saito M. Nakagawa M. Kaseda S. Matsuzaki T. Jonosono M. Eiraku N. Kubota R. Takenouchi N. Nagai M. Furukawa Y. Usuku K. Izumo S. Osame M. (2004). Decreased human T lymphotropic virus type I (HTLV-I) provirus load and alteration in T cell phenotype after interferon-alpha therapy for HTLV-I-associated myelopathy/tropical spastic paraparesis. *J. Infect. Dis.* 189 29–40 14702150 10.1086/380101

[B69] Satou Y. Yasunaga J. Yoshida M. Matsuoka M. (2006). HTLV-I basic leucine zipper factor gene mRNA supports proliferation of adult T cell leukemia cells. *Proc. Natl. Acad. Sci. U.S.A.* 103 720–725 16407133 10.1073/pnas.0507631103 PMC1334651

[B70] Satou Y. Yasunaga J. Zhao T. Yoshida M. Miyazato P. Takai K. Shimizu K. Ohshima K. Green P. L. Ohkura N. Yamaguchi T. Ono M. Sakaguchi S. Matsuoka M. (2011). HTLV-1 bZIP factor induces T-cell lymphoma and systemic inflammation *in vivo*. *PLoS Pathog.* 7 e1001274 10.1371/journal.ppat.1001274 PMC3037353 21347344

[B71] Sodroski J. Rosen C. Goh W. C. Haseltine W. (1985). A transcriptional activator protein encoded by the x-lor region of the human T-cell leukemia virus. *Science* 228 1430–1434 2990028 10.1126/science.2990028

[B72] Suemori K. Fujiwara H. Ochi T. Ogawa T. Matsuoka M. Matsumoto T. Mesnard J. M. Yasukawa M. (2009). HBZ is an immunogenic protein, but not a target antigen for human T-cell leukemia virus type 1-specific cytotoxic T lymphocytes. *J. Gen. Virol.* 90 1806–1811 19423550 10.1099/vir.0.010199-0

[B73] Sugata K. Satou Y. Yasunaga J. Hara H. Ohshima K. Utsunomiya A. Mitsuyama M. Matsuoka M. (2012). HTLV-1 bZIP factor impairs cell-mediated immunity by suppressing production of Th1 cytokines. *Blood* 119 434–444 22123848 10.1182/blood-2011-05-357459 PMC3257009

[B74] Suzuki S. Uozumi K. Maeda M. Yamasuji Y. Hashimoto S. Komorizono Y. Owatari S. Tokunaga M. Haraguchi K. Arima N. (2006). Adult T-cell leukemia in a liver transplant recipient that did not progress after onset of graft rejection. *Int. J. Hematol.* 83 429–432 16787875 10.1532/IJH97.05158

[B75] Tajima K. (1990). The 4th nation-wide study of adult T-cell leukemia/lymphoma (ATL) in Japan: estimates of risk of ATL and its geographical and clinical features. The T- and B-cell Malignancy Study Group. *Int. J. Cancer* 45 237–243 2303290 10.1002/ijc.2910450206

[B76] Takamori A. Hasegawa A. Utsunomiya A. Maeda Y. Yamano Y. Masuda M. Shimizu Y. Tamai Y. Sasada A. Zeng N. Choi I. Uike N. Okamura J. Watanabe T. Masuda T. Kannagi M. (2011). Functional impairment of Tax-specific but not cytomegalovirus-specific CD8 T lymphocytes in a minor population of asymptomatic human T-cell leukemia virus type 1-carriers. *Retrovirology* 8 100 10.1186/1742-4690-8-100 PMC3261825 22151736

[B77] Takeda S. Maeda M. Morikawa S. Taniguchi Y. Yasunaga J. Nosaka K. Tanaka Y. Matsuoka M. (2004). Genetic and epigenetic inactivation of tax gene in adult T-cell leukemia cells. *Int. J. Cancer* 109 559–567 14991578 10.1002/ijc.20007

[B78] Tamiya S. Matsuoka M. Etoh K. Watanabe T. Kamihira S. Yamaguchi K. Takatsuki K. (1996). Two types of defective human T-lymphotropic virus type I provirus in adult T-cell leukemia. *Blood* 88 3065–3073 8874205

[B79] Tangy F. Vernant J. C. Coscoy L. Ossondo M. Bellance R. Zaninovic V. Cartier L. Brahic M. Ozden S. (1995). A search for human T-cell leukemia virus type I in the lesions of patients with tropical spastic paraparesis and polymyositis. *Ann. Neurol.* 38 454–460 7668833 10.1002/ana.410380317

[B80] Tashiro T. Yamasaki T. Nagai H. Kikuchi H. Nasu M. (1992). Immunological studies on opportunistic infection and the development of adult T-cell leukemia. *Intern. Med.* 31 1132–1136 1421724 10.2169/internalmedicine.31.1132

[B81] Tattermusch S. Skinner J. A. Chaussabel D. Banchereau J. Berry M. P. Mcnab F. W. O’Garra A. Taylor G. P. Bangham C. R. (2012). Systems biology approaches reveal a specific interferon-inducible signature in HTLV-1 associated myelopathy. *PLoS Pathog.* 8 e1002480 10.1371/journal.ppat.1002480 PMC3266939 22291590

[B82] Tomaru U. Ikeda H. Ohya O. Abe M. Kasai T. Yamasita I. Morita K. Wakisaka A. Yoshiki T. (1996). Human T lymphocyte virus type I-induced myeloneuropathy in rats: implication of local activation of the pX and tumor necrosis factor-alpha genes in pathogenesis. *J. Infect. Dis.* 174 318–323 8699061 10.1093/infdis/174.2.318

[B83] Toulza F. Heaps A. Tanaka Y. Taylor G. P. Bangham C. R. (2008). High frequency of CD4+FoxP3+ cells in HTLV-1 infection: inverse correlation with HTLV-1-specific CTL response. *Blood* 111 5047–5053 18094326 10.1182/blood-2007-10-118539 PMC2602587

[B84] Uchiyama T. (1997). Human T cell leukemia virus type I (HTLV-I) and human diseases. *Annu. Rev. Immunol.* 15 15–37 9143680 10.1146/annurev.immunol.15.1.15

[B85] Yamano Y. Nagai M. Brennan M. Mora C. A. Soldan S. S. Tomaru U. Takenouchi N. Izumo S. Osame M. Jacobson S. (2002). Correlation of human T-cell lymphotropic virus type 1 (HTLV-1) mRNA with proviral DNA load, virus-specific CD8(+) T cells, and disease severity in HTLV-1-associated myelopathy (HAM/TSP). *Blood* 99 88–94 11756157 10.1182/blood.v99.1.88

[B86] Yamazaki H. Ikeda H. Ishizu A. Nakamaru Y. Sugaya T. Kikuchi K. Yamada S. Wakisaka A. Kasai N. Koike T. Hatanaka M. Yoshiki T. (1997). A wide spectrum of collagen vascular and autoimmune diseases in transgenic rats carrying the env-pX gene of human T lymphocyte virus type I. *Int. Immunol.* 9 339–346 9040015 10.1093/intimm/9.2.339

[B87] Yoshida M. (2001). Multiple viral strategies of HTLV-1 for dysregulation of cell growth control. *Annu. Rev. Immunol.* 19 475–496 11244044 10.1146/annurev.immunol.19.1.475

[B88] Younis I. Green P. L. (2005). The human T-cell leukemia virus Rex protein. *Front. Biosci.* 10 431–445 15574380 10.2741/1539 PMC2659543

[B89] Zhang J. Yamada O. Kawagishi K. Araki H. Yamaoka S. Hattori T. Shimotohno K. (2008). Human T-cell leukemia virus type 1 Tax modulates interferon-alpha signal transduction through competitive usage of the coactivator CBP/p300. *Virology* 379 306–313 18678383 10.1016/j.virol.2008.06.035

[B90] Zhao T. Satou Y. Sugata K. Miyazato P. Green P. L. Imamura T. Matsuoka M. (2011). HTLV-1 bZIP factor enhances TGF-beta signaling through p300 coactivator. *Blood* 118 1865–1876 21705495 10.1182/blood-2010-12-326199 PMC3158717

